# Financing and current capacity for REDD+ readiness and monitoring, measurement, reporting and verification in the Congo Basin

**DOI:** 10.1098/rstb.2012.0310

**Published:** 2013-09-05

**Authors:** Danae Maniatis, Jérôme Gaugris, Danilo Mollicone, Joel Scriven, Alexis Corblin, Cleto Ndikumagenge, André Aquino, Philippe Crete, Maria-José Sanz-Sanchez

**Affiliations:** 1 Forestry Department, UN-REDD Programme, Food and Agriculture Organization of the United Nations, Rome, Italy; 2 School of Geography and the Environment - Environmental Change Institute, University of Oxford, Oxford, UK; 3 Centre for Wildlife Management, University of Pretoria, Pretoria, South Africa; 4 Africa Unit - Forest Carbon Partnership Facility, World Bank, Washington, DC, USA

**Keywords:** REDD+ readiness, national forest monitoring systems, measurement, reporting and verification, Central African Commission, financing

## Abstract

This paper provides the first critical analysis of the financing and current capacity for REDD+ readiness in the Congo Basin, with a particular focus on the REDD+ component of national forest monitoring and measurement, reporting and verification (M&MRV). We focus on three areas of analysis: (i) general financing for REDD+ readiness especially M&MRV; (ii) capacity and information for REDD+ implementation and M&MRV; (iii) prospects and challenges for REDD+ and M&MRV readiness in terms of financing and capacity. For the first area of analysis, a REDD+ and M&MRV readiness financing database was created based on the information from the REDD+ voluntary database and Internet searches. For the second area of analysis, a qualitative approach to data collection was adopted (semi-structured interviews with key stakeholders, surveys and observations). All 10 countries were visited between 2010 and 2012. We find that: (i) a significant amount of REDD+ financing flows into the Congo Basin (±US$550 million or almost half of the REDD+ financing for the African continent); (ii) across countries, there is an important disequilibrium in terms of REDD+ and M&MRV readiness financing, political engagement, comprehension and capacity, which also appears to be a key barrier to countries receiving equal resources; (iii) most financing appears to go to smaller scale (subnational) REDD+ projects; (iv) four distinct country groups in terms of REDD+ readiness and M&MRV status are identified; and (v) the Congo Basin has a distinct opportunity to have a specific REDD+ financing window for large-scale and more targeted national REDD+ programmes through a specific fund for the region.

## 1. Introduction

**Figure 1.**
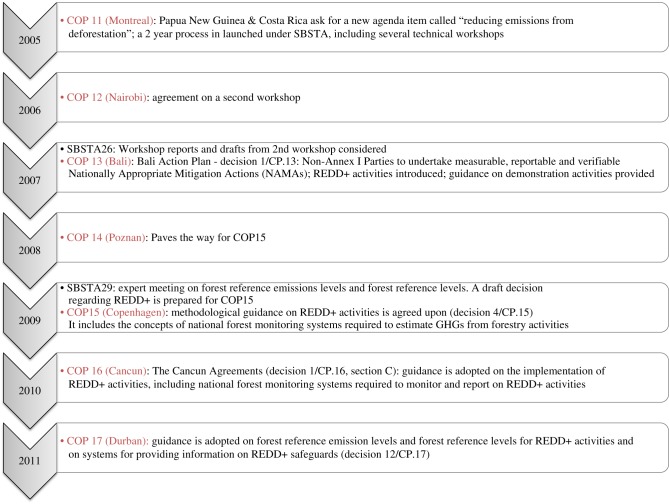
Progress of REDD+ discussions and key decisions relating to REDD+ and M&MRV from COP11 to COP17.

As part of international climate change mitigation efforts, in the context of the implementation of the United Nations Framework Convention on Climate Change (UNFCCC), developing countries are encouraged to reduce greenhouse gas (GHG) emissions from deforestation and forest degradation, conserve and sustainably manage their forests and enhance forest carbon stocks, referred to as REDD+ activities^1^. The broad scope of REDD+ was agreed upon in recognition of different countries' circumstances, to promote broad country participation. Negotiations on REDD+ can be traced to the 11th session of the UNFCCC Conference of the Parties (COP) [1], where ‘reducing emissions from deforestation in developing countries’ was raised as an agenda item under the COP [1] that initiated a two year work programme under the UNFCCC's Subsidiary Body for Scientific and Technological Advice (SBSTA). Figure 1 provides a timeline of UNFCCC discussions on REDD+ [2–6].

To prepare for the implementation of the REDD+ activities at the national level, the UNFCCC encourages developing countries to follow a three-phased approach: (i) readiness, (ii) demonstration activities and policy implementation as well as (iii) positive incentives for results-based action leading to develop the necessary technical and institutional capacities and systems. The ‘REDD+ readiness’ phase is the period of capacity-building required prior to full national implementation. It includes the preparation of a national REDD+ strategy and its legal and institutional implementation framework, development of national forest reference emission levels and/or forest reference levels and the implementation of a national forest monitoring system that includes a GHG measurement, reporting and verification (M&MRV) system.

An important challenge for REDD+ readiness is financing. Economic analyses by Stern [7] and Eliasch [8] suggest that REDD+ could be a cost-effective, low-tech, inclusive and quick way to contribute to mitigation, by putting a value on forest carbon [9]. However, following a number of years’ experience in REDD+ readiness financing, it seems increasingly unclear whether carbon-related revenues will be able to compete with revenues from other land uses, such as conversion of forest land for agricultural production to meet global fuel and food demands [10]. This is especially important for the Congo Basin where the majority of forests have been spared the pressure of large-scale conversions, although this is likely to change as land conversion associated with economic development progresses in the near future. This is already the case for oil palm plantations that have been established in some of the Congo Basin's countries [11].

The total financing that has been disbursed and committed for REDD+ readiness is challenging to establish, but a current estimate reported by funders through the voluntary REDD+ database indicates a figure of approximately US$ 6.06 billion since 2006 (*Redd+ online voluntary database*: http://reddplusdatabase.org/), with REDD+ readiness financing to beneficiary countries on the African continent totalling approximately US$1 billion (*Redd+ online voluntary database*: http://reddplusdatabase.org/). One of the main challenges the REDD+ process (and the provision of adequate and timely capacity-building support) faces today, is to understand how much financing is being invested in REDD+ readiness. What is becoming increasingly apparent is that although the UNFCCC REDD+ process is based on the principle of ‘inclusiveness’ (i.e. all countries wishing to voluntarily participate in the process should be able to access ‘support’—including financing for readiness) [5],^2^ support to developing countries has been uneven. In addressing this disparity, the international community should seek to ensure that ‘equal’ opportunities are provided to developing countries wishing to participate in REDD+ readiness, while ensuring that financing is provided where it is believed that it will have the most impact (i.e. balancing efficiency and inclusiveness). By taking an analytical approach to evaluating existing financing for REDD+ M&MRV readiness and existing REDD+ M&MRV capacity, our analysis highlights where such disparities exist in the Congo Basin.

The objective of this paper is to provide a critical analysis of the progress and challenges for REDD+ readiness in terms of financing and capacity, with a particular focus on M&MRV, in the Congo Basin. To do so, we focus on three areas of analysis: (i) general financing for REDD+ readiness and specifically M&MRV readiness; (ii) capacity and information for REDD+ readiness and M&MRV currently existing in the countries and; (iii) prospects and challenges for REDD+ and M&MRV readiness in terms of financing and capacity.

### (a) The Congo Basin

The geographical focus of this article is Africa's Congo Basin, which covers the 10 Central African Commission (COMIFAC) countries: Burundi, Cameroon, Central African Republic (CAR), Chad, Republic of the Congo (RoC), Democratic Republic of the Congo (DRC), Equatorial Guinea (EG), Gabon, Rwanda, São Tomé and Principe (STP). These countries have a combined forest area (moist and dry) of 293 million ha ±25 million ha and historically low rates of forest loss (deforestation) [12]. Large primary forest blocks still remain in DRC, Gabon and RoC, with DRC holding 63% of total remaining forest [13]. Although the causes and drivers of deforestation in the region are extremely complex and differ between countries, the most commonly cited proximate cause of deforestation in the COMIFAC countries is the expansion of subsistence activities (shifting agriculture, small-scale permanent agriculture and firewood collection) [14]. However, increasing pressure from a variety of sources can be observed, including hydrocarbon extraction and opening of new mines [15], agribusiness, road development, biofuels and charcoal collection [14].

In this regional context, the objective of this paper is to provide a critical analysis of the progress and challenges for REDD+ readiness in terms of financing and capacity, with a particular focus on M&MRV.

### (b) Forest monitoring and measurement, reporting and verification

Initial methodological guidance for M&MRV for REDD+ was provided at the 15th COP (COP 15) held in Copenhagen (December 2009), making these relatively new concepts for developing countries to grapple with.

Since then, the scientific literature has sought to decipher the meanings and applications of the concepts and terminology of monitoring, measurement, reporting and verification in the REDD+ context, without an overall consensus being reached to date. Generally speaking, UNFCCC decisions either refer to ‘monitoring and reporting’ or ‘measurement, reporting and verification’. Therefore, although no ‘exact’ definitions have been provided for these terms under the UNFCCC, it is clear that policy makers have paid great attention to this terminology and the implications they have. In UNFCCC jargon, ‘monitoring’ is often regarded as the need for periodic information on the results obtained through national policies and measures, as per Article 4.2, paragraphs (a) and (b) of the Convention [16].^3^ ‘MRV’, on the other hand, can be interpreted as the means to address countries’ commitments to collect and share information on their national GHG emissions and removals under Article 4.1 (a) of the Convention [16].^4^

It was during COP15 in Copenhagen that the concept of ‘monitoring and reporting’ was used for REDD+ [4, paragraph 1], though it was not until the 16th COP in Cancun that ‘measurement, reporting and verification’ was introduced [5, paragraph 73], linking REDD+ to a future financial mechanism. For REDD+, ‘monitoring and reporting’ is applied throughout phases 2 and 3, while ‘MRV’ is only applicable in phase 3—when it will be a pre-condition of results-based positive incentives (such as finance) [5]. Based on the above and to avoid any confusion, we use the terminology of M&MRV in this paper.

## 2. Methodology

To evaluate the status, prospects and challenges for REDD+ financing readiness and M&MRV in the Congo Basin, a team of four UN-REDD M&MRV experts^5^ working for the Food and Agricultural Organization (FAO) visited the 10 COMIFAC countries during 2010–2012. This process was undertaken for the development of a COMIFAC project on M&MRV, funded by the Congo Basin Forest Fund, carried by the Executive Secretariat of the COMIFAC, with FAO as the executing agency and the Brazilian Space Agency (INPE) as a technical partner.

Bilateral and multilateral meetings were held with REDD+ donors involved in the region (the governments of Norway and United Kingdom), banks (the African Development Bank, the World Bank and Global Environmental Facility (GEF)) and funds (the Congo Basin Forest Fund—CBFF) and other financial and technical CBFP partners. Three regional workshops were held, each having approximately the same composition of actors as specified above: Douala (Cameroon) 2010, Bujumbura (Burundi) 2011 and N'Djamena (Chad) 2012.

### (a) REDD+ and REDD+ measurement, reporting and verification readiness financing database

A database was created for this paper to facilitate the evaluation of REDD+ financing in each of the 10 countries as well as at the regional level. This was done in two steps. First, the main input into this database was the ‘raw data’ of the REDD+ voluntary database, dated August 2012. Each entry in the REDD+ voluntary database was analysed for relevant financing for REDD+ in the COMIFAC area since 2006 and inserted in the database developed for this paper. It should be noted that the REDD+ voluntary database does not include sub-national geographically explicit information on where REDD+ projects are being implemented within the countries. Furthermore, as the REDD+ voluntary database depends on the voluntary contributions made by participants, it is currently very difficult to distinguish between financing that has been committed and financing that has been disbursed. For the purposes of this paper, we do not draw this distinction, and use the overall term of financing that has been ‘committed’ (knowing that some of it has been disbursed and received, but we are unable to specify how much and/or when). To date, no information has been submitted by private sector entities to the REDD+ voluntary database. Private sector entities are therefore not taken into account in this analysis.

The second step was to complement and crosscheck the entries retained from the above first step of analysis. A number of additional information sources were thereby accessed: UN-REDD Programme, Forest Carbon Partnership Facility (FCPF), ITTO-REDDES, the REDDdesk and various other results from Internet searches using the Google search engine and the following key words in French and English: ‘REDD+’, ’REDD’, ‘finance’, ‘investment’, ‘reducing emissions from deforestation’. Search results were checked and filtered for relevant information. Additional results from Internet searches were added to the database for this paper as ‘entries’ (individual financing lines). Only financing targeted at a specific country was listed at the country level (including sub-national financing); all other financing was listed under ‘regional’ financing. The database collected information on: project name, financing amount, donor, financing modalities, financing type, objective, REDD+ action categories, M&MRV related, recipients, recipient types (non-governmental organizations, NGOs/government/private/inter-governmental organizations, IGOs/research), beneficiary countries, additional info, from–to dates and source.

### (b) REDD+ measurement, reporting and verification capacity assessment

During in-country visits, a qualitative approach to data collection was adopted (semi-structured interviews with key stakeholders, surveys and observations—see electronic supplementary material S1) in order to assess the existing institutional and technical situations in terms of REDD+ and M&MRV. Meetings were held with a range of stakeholders: governments, REDD+/UNFCCC climate change focal points, IGOs, NGOs, regional initiative's representation offices, civil society representatives and research institutes. Stakeholders were identified through preparatory meetings with government officials, the Executive Secretariat of the COMIFAC and UN-REDD. Unfortunately, the team was unable to meet with the few REDD+ project developers, who were operational in the COMIFAC countries during 2010–2012. Pre-prepared sheets were filled in through semi-structured interviews and observations together with stakeholders on various aspects of REDD+ and M&MRV (comprehension, status, on-going activities, etc*.*).

Three key components of REDD+ M&MRV were considered during the semi-structured interviews, surveys and observations: (I) a national GHG-inventory; (II) a national forest inventory and (III) a satellite forest representation system. For each of these components, countries were assessed by experts on seven elements: (a) existing inventory; (b) basic information; (c) agent's level of expertise; (d) level of training in country; (e) availability of premises; (f) availability of material and (g) communication level (Internet/telephone). Each of these elements (a–g) was given a score for each of the three (I–III) M&MRV components, ranging from 1 to 3: 1, low capacity; 2, average capacity and 3, advanced capacity (table 1). The maximum score for one of the three M&MRV components is therefore 21 (maximum score of three for each of the seven elements) and for a country 63 (each of the three key components (I–III)×the maximum score of the seven elements 21).

**Table 1.** Definition of low, average and advanced capacity for each of the seven elements (a–g) against which countries were assessed for each of the three M&MRV components: (I) a national GHG inventory; (II) a national forest inventory and (III) a satellite forest representation system.

capacity	(a) inventory	(b) basic information	(c) level of expertise	(d) level of training	(e) availability of premises	(f) availability of material	(g) communication level (Internet/telephone)
low	not started/not operational/in design phase	no—little information available/no centralization of information	no to little understanding (subject mostly new)	none—very little received/unable to apply	premises are not entirely secure/no generators/not entirely suitable for the analysis laboratory of the M&MRV component in question/institutions unable to put premises at disposal	most materials needed for operationalization are absent	not in place/not functional
average	partially being implemented/completed	some information available/some centralization of information	some understanding but notable gaps/partially able to apply	some received/partially able to apply	premises are more or less secure/electricity most of the time/generator working some of the time/institutions would be able to put premises at disposal if premises improved considerably	some materials needed for operationalization acquired/some important materials not acquired/no real inventory of existing materials	partially in place and functional
advanced	complete or in final stages of completion	good and relevant information available/advanced centralization of information	good understanding/mostly able to apply	targeted training received	premises are secure/electricity functional/working generator/institutions able to put premises at disposal with little additional improvements	most materials needed for operationalization already acquired/relatively complete inventory of existing materials	in place and mostly functional

### (c) Prospects and challenges for REDD+ readiness and REDD+ measurement, reporting and verification capacity

During the M&MRV kick-off meeting of the regional M&MRV project in N'Djamena (September 2012), three working groups were formed: one on the country programmes, one on the regional programme and one on the technical assistance programme. Each of the groups comprised 10–20 people, representing governments, NGOs, IGOs and civil society and was asked, among other things, to identify the key prospects and challenges for REDD+ and M&MRV in the Congo Basin.

## 3. The current status of readiness for REDD+ and M&MRV in the Congo Basin

### (a) Readiness financing for REDD+ and measurement, reporting and verification

This section evaluates the results of the assessment of the database built for this paper, totalling 129 evaluated entries.

#### (i) Participation in global REDD+ readiness financing initiatives

Table 2 shows the participation in global REDD+ readiness initiatives at national and regional levels. The table illustrates a clear disparity in financing received to date by the 10 countries. Four high forest cover countries (Cameroon, RoC, DRC and Gabon) participate in most of the international initiatives and receive direct financing. Meanwhile, low forest cover (LFC) countries are not engaged in the process to the same extent. Equatorial Guinea is singled out as a high forest cover country with little engagement in any major REDD+ readiness financing.

**Table 2.** Participation in global initiatives in REDD+ readiness financing (✗= not receiving any financing; ✓ = part of the programme but not receiving any direct financing from these programmes; ✓$ = receiving direct financing). The last column ‘total for all financing received’ adds up all of the financing per country and on the regional level. Abbreviations: CAR, Central African Republic; RoC, Republic of the Congo; DRC, Democratic Republic of the Congo; EG, Equatorial Guinea; STP, São Tomé and Principe.

region/country	UN-REDD	FCPF	FIP	GEF	bilateral	CBFF	ITTO-REDDES	total for all financing committed (in $)
COMIFAC	✗	✗	✗	✓$	✓$	✓$	✗	189 176 00
Burundi	✗	✗	✗	✓$	✗	✓$	✗	4 374 000
Cameroon	✗	✓$	✗	✓$	✓$	✓$	✓$	80 870 832
CAR	✓	✓$	✗	✓$	✗	✓$	✗	5 148 000
Chad	✗	✗	✗	✗	✗	✓$	✗	0
RoC	✓$	✓$	✗	✓$	✓$	✓$	✗	12 126 000
DRC	✓$	✓$	✓$	✓$	✓$	✓$	✓$	227 876 100
EG	✗	✓	✗	✗	✗	✓$	✗	0
Gabon	✓	✓	✗	✗	✓$	✓$	✗	22 368 974
Rwanda	✗	✗	✗	✗	✓$	✓$	✗	8 360 000
STP	✗	✗	✗	✗	✗	✓$	✗	0
	$11 383 000	$7 399 100	$60 250 000	$58 599 000	$209 902 000	$117 651 999	$450 000	550 125 906

#### (ii) Financial flows to the region

With national and regional initiatives combined, ±US$550 million has been disbursed or committed towards the COMIFAC area to support REDD+ readiness and projects since 2006. Of this, ±US$189 million was received at the ‘regional’ level (multi-country financing) and approximately ±US$361 million at the national level. Three countries have received no direct national REDD+ readiness financing to date: Chad, Equatorial Guinea and STP. The largest percentage of financing was allocated to DRC (41%), followed by the regional level (34%), Cameroon (15%), Gabon (4%), RoC (2%), Rwanda (2%), Burundi (1%) and CAR (1%).

According to table 2, it appears that countries that are part of REDD+ readiness financing initiatives receive ‘more’ financing than countries that are not part of such initiatives.

#### (iii) Financing modalities

At the national level (table 3), most financing modalities appear to be multilateral,^6^ while at the regional level (table 4) multilateral and bilateral^7^ financing modalities are almost of equal importance.

**Table 3.** Financing modalities on the national level.

national level	number of entries	financial sum (in $)
multilateral	48	185 853 099
bilateral	23	107 069 000
private	2	4 200 000
no information	23	71 001 807
total	96	368 123 906

**Table 4.** Financing modalities on the regional level.

regional level	number of entries	financial sum (in $)
multilateral	17	78 039 000
bilateral	11	80 175 000
bilateral and multilateral	1	7 658 000
bilateral, domestic	1	15 000 000
no information	4	6 040 000
total	35	189 002 000

Bilateral financing is flowing primarily to high forest cover countries: Cameroon (±US$25 million), DRC (±US$79 million); and to a lesser extent Gabon (±US$80 000) and RoC (±US$80 000), with Rwanda (±US$2 million) being the exception. Within the above-mentioned, Cameroon has eight entries, DRC has 12, and Gabon, RoC and Rwanda have one each—illustrating the greater levels of bilateral financing being received by DRC and Cameroon.

At the multilateral level, the Congo Basin Forest Fund and Global Environmental Facility represent the main regional sources for REDD+ readiness financing, totalling approximately ±US$117.7 million and ±US$58 million, respectively. The Forest Investment Programme made a single investment of ±US$60 million in DRC; the FCPF committed around US$7.3 million, while the UN-REDD Programme has financed approximately US$12 million.

The Congo Basin Forest Fund is the only financing source directly targeting the ten COMIFAC countries, funding ±US$37.1 million at the regional level (i.e. multi-country projects) and ±US$80.4 million directly at the national level (in-country projects). It is notable that 45% of funds to date have been directly invested in DRC.

#### (iv) Recipients

Evaluating the financing that is committed at the national level (±US$361), 77% of it has been committed directly to governments (±US$277.3 million), while 17% is committed to NGOs (±US$61.8 million), and 2% to private entities (±US$8.3 million), IGOs (±US$7.5 million) and a combination of other (state and non-state) stakeholders (±US$5.9 million).

Looking at financing channelled through the regional level alone, a substantial portion (37%) of the total financing (±US$189 million) is committed directly to NGOs (±US$69.5 million), 21% to a combination of stakeholders (±US$39.3 million), 9% to the Executive Secretariat of COMIFAC and governments simultaneously (±US$17.9 million), 9% to research institutions (±US$16.7 million) and 8% to the Executive Secretariat of COMIFAC. Only 1% is committed directly to governments when financing is of a multi-country nature. The other multilateral financing types are: 5% unidentified (±US$10.3 million), 2% a combination of IGOs and governments (±US$4.7 million), 2% to the Joint Research Centre of the European Commission (±US$3.7 million), 2% research and NGO combined (±US$3.6 million), 2% to foundations (±US$3 million) and 1% to IGOs (±US$2.6 million).

#### (v) Financing for M&MRV

There are 10 entries (projects) at the regional level related to M&MRV. Although this totals ±US$75 million, only four of these entries are strictly related to M&MRV, totalling ±US$26.3 million, which can be considered to be the most conservative REDD+ readiness financing estimate targeted specifically at M&MRV at the regional level.

At the national level, 24 entries related to M&MRV total US$105.4 million. However, as with the regional level, only seven of these entries are strictly related to M&MRV. A conservative estimate for REDD+ M&MRV readiness financing at the national level is±US$22.1 million (with±US$10.2 million in DRC; ±US$4.3 million in RoC and ±US$7.6 million in Gabon).

### (b) Readiness for REDD+ and measurement, reporting and verification related to capacity and information

This analysis provides a first insight into where countries currently find themselves in this process; and as such does not account for the fact that (i) countries started with varied capacities for M&MRV prior to engaging in REDD+ readiness and (ii) countries entered (or have not yet entered) the REDD+ readiness process at different points in time.

Based on the semi-structured interviews and observations, table 5 provides an overview of the status of comprehension, active national programmes and NGO involvement in the REDD+ process for each of the 10 countries. ‘Comprehension’ was measured based on elements of REDD+ in terms of: the concept of REDD+; understanding of the UNFCCC negotiations, processes and decisions texts; and knowledge of international programmes to support REDD+ readiness such as UN-REDD and FCPF.

**Table 5.** Understanding of REDD+ and overall REDD+ status in the countries.

	comprehension	national programmes	NGOs
Burundi	concept is inadequately understood	no national REDD+ programme exists	no NGOs REDD+ capacity, although some NGOs have a reasonable understanding of the importance of REDD+ for the country
Cameroon	concept is gaining understanding in the country, creation of a climate change office under the Prime Minister's office	R-PP approved	NGO REDD+ capacity and activity exists
CAR	concept needs to gain understanding in the country national REDD+ Committee, REDD+ Technical Committee and REDD+ inter-prefectural REDD+ created	project for the Support to the Implementation of Forest Management Plans (PARPAF)	little NGO REDD+ capacity
Chad	very new concept	a few national programmes such as the Green Belt around N'Djamena project	very few NGOs work in this sector
RoC	a concept that is fairly well understood with various initiatives going on at the national level; existing national REDD+ committee and REDD+ coordination	R-PP approved REDD+ projects are being developed	there is an active NGO REDD+ capacity and involvement
DRC	a concept that has acquired much understanding in the country already	several programmes and projects, notably the FIP	active NGO involvement
	national REDD+ Committee and REDD+ inter-ministerial committee created	REDD+ projects are operational on the field	
EG	fairly new concept for the country very few stakeholders know what this term and its implications mean	no information	few NGO's work on this topic
Gabon	concept that is gaining understanding in the country with various initiatives going on at the national level	development of the climate plan	there is an active NGO REDD+ capacity and involvement
Rwanda	concept vaguely understood	a 50% country cover NFI is currently being developed	no direct NGOs involvement in REDD+; however, many local NGOs are involved in forest related activities
STP	concept vaguely understood	no information	many local NGOs are involved in activities related to forests and could be easily associated with REDD+

Half of the countries have engaged, to varying degrees, in the first phase (readiness) of REDD+ at the national level: Cameroon, CAR, RoC, DRC and Gabon. Gabon is the only country integrating REDD+ as one of several mitigation actions into a national ‘Climate Change Plan’, incorporating it with its sustainable development strategy.

In terms of levels of comprehension of the REDD+ process and implementation of the readiness phase, DRC is the most advanced, having completed major components of its readiness plan (initiated in 2009), a mid-term review of its process, and hosting several on-going demonstration projects. RoC obtained funding from FCPF and the UN-REDD Programme in 2012 to implement its readiness preparation proposal (RPP) and is actively engaging donors to operationalize and finance REDD+ demonstration activities. CAR did not update their FCPF RPP taking into account comments of the FCPF Participant's Committee and has therefore not yet received any funding. Cameroon's RPP was approved in October 2012 by the FCPF Participant's Committee.

Burundi, Chad, Equatorial Guinea, Rwanda and STP are less engaged in the REDD+ process, although, of these, Chad and Burundi appear more advanced. Although the NGO Conservation International initiated an international interest in REDD+ in Equatorial Guinea, the process does not feature high on the country's political agenda, and the country lost its membership to the FCPF for not signing the Participation Agreement. Rwanda has not engaged in the REDD+ process, though there is a strong interest at the national level to do so. STP appears to be the least engaged in the process.

Relating the above to the section on REDD+ financing, this analysis suggests that four of the five countries having received most of the financing for REDD+ readiness are indeed advanced in the REDD+ readiness process in terms of their comprehension of REDD+: DRC, Cameroon, Gabon and RoC (the exception being CAR).

Table 6 presents the status of M&MRV in the 10 countries and considers three key components of M&MRV: a national GHG-inventory, a national forest inventory and a satellite forest representation system. The average score of the 10 countries is 33, indicating median M&MRV capacity. Three distinct groups of countries can be distinguished in terms of M&MRV capacity. The first group comprises DRC, Gabon and Rwanda with a higher than average assessed capacity. The second group includes RoC and Cameroon with a slightly above average scoring and finally a third group with below average scoring including Burundi, Chad, CAR, Equatorial Guinea and STP.

**Table 6.** M&MRV status in countries. 1, low; 2, average and 3, advanced. The scoring on the right-hand column ‘score’ indicates the score per M&MRV component, while the scoring under each country indicates the total scoring (out of 63) of the country in terms of the M&MRV assessment.

	M&MRV	inventory	basic information	agent's level of expertise	level of training	availability of premises	availability of material	communication level (Internet/telephone)	score
Burundi 28	GHG-I	2	2	2	1	1	1	1	10
	NFI	1	1	2	1	1	1	1	8
	SRTS	2	2	2	1	1	1	1	10
Cameroon 34	GHG-I	2	2	2	2	1	1	1	11
	NFI	3	3	2	2	1	1	1	13
	SRTS	1	3	1	2	1	1	1	10
CAR 22	GHG-I	2	1	1	1	1	1	1	8
	NFI	1	1	1	1	1	1	1	7
	SRTS	1	1	1	1	1	1	1	7
Chad 29	GHG-I	2	2	2	2	2	1	2	13
	NFI	1	1	1	1	1	1	1	7
	SRTS	1	1	1	2	1	1	2	9
RoC 36	GHG-I	3	2	2	1	1	1	1	11
	NFI	3	3	2	2	1	2	2	15
	SRTS	1	2	1	2	1	2	1	10
DRC 44	GHG-I	3	2	2	1	2	2	2	14
	NFI	1	1	2	1	2	3	2	12
	SRTS	3	2	2	2	3	3	3	18
Gabon 42	GHG-I	2	2	2	2	2	2	2	14
	NFI	1	3	2	2	2	2	2	14
	SRTS	2	2	2	2	2	2	2	14
EG 27	GHG-I	1	1	2	2	3	1	1	11
	NFI	1	1	1	1	2	1	1	8
	SRTS	1	1	1	1	2	1	1	8
Rwanda 42	GHG-I	2	2	2	2	2	1	2	13
	NFI	2	2	2	3	2	1	2	14
	SRTS	2	2	3	3	2	1	2	15
STP 26	GHG-I	2	2	2	1	1	2	1	11
	NFI	2	1	1	1	1	1	1	8
	SRTS	1	1	1	1	1	1	1	7

In terms of M&MRV financing and the status assessment in the countries, the relationship appears to be less straightforward than for the general REDD+ process. The three countries that have received direct M&MRV financing at the national level, as identified previously, are DRC, RoC and Gabon. Nonetheless, Rwanda finds itself in the ‘most advanced’ group, while RoC finds itself in the ‘average level’ group together with Cameroon, which received no direct M&MRV financing.

### (c) Challenges and prospects

One of the tasks set for each of the working groups during the workshop in N'Djamena in September 2012 was to identify the key prospects and challenges to implement REDD+ and particularly M&MRV within their group.

The working groups identified three main challenges. First, more balanced financing opportunities to enable each country to enter the REDD+ readiness phase (e.g. by preparing and submitting an RPP) and a more inclusive approach to the mobilization of funds were identified as challenges at both national and regional levels. The perception in the working groups was that primarily a lack (or inability) of access to financing by some countries is the major barrier to readiness implementation. Second, country ownership of the REDD+ readiness process and M&MRV, as well as access to information related to these two concepts, were also identified as challenges. This is related to a lack of understanding of the COP decisions and IPCC guidance among countries hindering their engagement and adequate activity implementation. The key observation made was a feeling of a lack of ownership owing to (i) a lack of understanding the existing UNFCCC Decisions on REDD+; (ii) the impression that the readiness phase at the national level can be driven more by outside intervention of IGOs than by national processes; and (iii) that readiness projects at the sub-national scale (often driven by NGOs) are rarely connected to the national process. Third, coordination between international technical partners was also identified as a key challenge, especially relating to M&MRV. The point was made that in countries where international technical partners support the national government, these often face challenges in coordinating readiness financing, methods, etc. and that the danger of competition between technical partners exists, sometimes bringing about confusion among national counterparts. It was also pointed out that even the technical partners were at times struggling to understand and grapple with readiness for M&MRV as these were new concepts in forest management and REDD+, and ones on which no international consensus has been reached. DRC was put forward as an example where technical partners took the time to work on a single, complementary approach to M&MRV readiness at the national level.

In addition to the challenges, the working groups also identified four key prospects. First, country engagement and leadership in REDD+ implementation and effective engagement of regional and national stakeholders in the REDD+ process is a current key strength. ‘Engagement’ was discussed in terms of decision-making power, ability to mandate and mobilize institutions and stakeholder consultations (where the stakeholders had a real comprehension of the process). Developing national expertise through REDD+ and M&MRV readiness financing and targeted capacity building was identified as a second prospect. Third, knowledge sharing and transnational lesson learning (i.e. from the more ‘advanced’ countries in the process) was also identified as an important opportunity. Some projects and countries have started inviting climate change focal points from more ‘advanced’ countries in the REDD+ and M&MRV readiness phase to workshops to share their experiences. Finally, the possibility of having in place a specific regional financing window, for example, the Congo Basin Forest Fund, was identified as an important prospect for strategically targeting specific components of REDD+ readiness financing and capacity gaps and putting in place the formal procedures to access REDD+ readiness financing more tailored to COMIFAC countries’ needs.

## 4. Discussion

### (a) REDD+ and REDD+ measurement, reporting and verification financing

A considerable amount of REDD+ (±US$550 million) and M&MRV (±US$26–75 million) readiness financing has been committed to the COMIFAC region since 2006 (when the process was initiated at the international level). These figures should be considered approximate, as we believe that more financing might have been made available through more diffuse sources. Furthermore, as the main data source used is the REDD+ voluntary database, it is important to note that we were unable to place projects or funding related to M&MRV that are not specifically REDD+ related but could have relevance for M&MRV, especially with respect to human capacity building, strengthening of institutional capacity through the provision of equipment and operational support, etc. Nonetheless, for REDD+, ±US$550 million is almost double the figure previously estimated in early 2012 (±US$287 million) [17]. This could partially be because COMIFAC [17] did not consider specialized country-level REDD+ financing mechanisms (e.g. programmes, such as UN-REDD and FCPF). An example of how monitoring of REDD+ financing and projects could be improved can be taken from DRC, which is implementing a registry to track overall financing to REDD+ in the country.

We observe a striking disequilibrium in terms of REDD+ and M&MRV financing, political engagement, fundamental comprehension and technical and institutional capacities in the COMIFAC region. Although country needs are different, there has clearly been a disparate approach to financing REDD+ readiness and M&MRV capacity building. This is a point of concern if REDD+ is to be inclusive and successful in the COMIFAC area. Though certain countries have expressed the political will to engage in readiness for REDD+ (such as, for example, Burundi and Chad), a number of obstacles remain. First, it is at the discretion of the multilateral funding bodies such as FCPF and the UN-REDD Programme to allocate those funds to a specific country and/or a specific activity. In other words, countries may be committed to enter the REDD+ readiness process and request support for REDD+ readiness funds, but be unable to obtain them. It should also be noted that FCPF and the UN-REDD Programme have limited resources, which further complicates the ability to respond adequately to country requests. Second, countries with lower institutional capacity might find it more difficult to engage with partners and procedures to access REDD+ readiness funds. It is clear from the challenges identified by the working groups that this non-inclusive financing approach and lack of understanding as to why this is happening are regarded as perhaps the most important current challenges for REDD+ at both national and regional levels. What remains to be seen is how country financing can be tackled proportionally (e.g. forest extent, emissions originating from the forest sector, existing capacity, etc.).

In the COMIFAC countries, where political institutions are often weak and capacity low, a blanket, rather complex approach for accessing REDD+ readiness financing (often at a scale of around US$ 3–4 million with many validation steps, e.g. R-PP process), may not be the most appropriate strategy to engage countries. Although not explicitly identified as a challenge in the working groups, we believe that developing a more strategic approach to dispersing financing by key multi-lateral partners and clearer communication to beneficiary countries on how to access financing, will be required to improve the process. There are two immediate obstacles to this: first, how do financing bodies assess whether or not the necessary political will is in place to initiate REDD+ readiness financing support? It would appear counter-productive to create a set of ‘indicators’ for this purpose, for example. Second, as can be observed from table 5, countries such as Burundi, Chad, Equatorial Guinea, Rwanda and STP appear to have a vague understanding of the REDD+ concept. However, this does not mean that they are not interested in engaging in the process; it instead indicates that they are unclear about how to engage. A possible way forward to dealing with these two obstacles is that REDD+ readiness financing could be initiated with a more limited scope and very specific institutional and capacity-building activities (e.g. awareness raising on REDD+ and M&MRV concepts, institutional arrangements, etc.), thereby supporting the country in question to understand if and how they would like to engage in REDD+ readiness (linking back to the challenge of access to information identified in the working groups). If such ‘targeted support’ is successful and the country in question and technical/political partners have demonstrated that results were achieved in this preliminary phase, then more comprehensive REDD+ readiness financing could follow. This process could be a way forward to allow countries with lower capacity to more easily enter a step-wise approach to REDD+ readiness financing and simultaneously allow financing bodies to provide similar levels of funds in a more inclusive and secure way.

On the other hand, countries with more political engagement and capacity, such as Gabon, are also facing difficulties in accessing REDD+ readiness financing, albeit owing to very different reasons. Gabon is currently the only country that is integrating REDD+ into a ‘National Climate Change Plan’, incorporating it with its low-carbon sustainable development strategy. This plan adopts a holistic approach by integrating land-use, exploitation of natural resources and GHGs, forests being only one part of the picture. The Gabonese Presidency mandates Gabon's political emergence in this field, endeavouring to implement a vision based on the country's human potential and natural resources (including minerals). Gabon is currently the only country in the region where the commitment of tackling climate change comes from the highest political level. In doing so, Gabon has chosen to adopt its own approach to address these questions, not adhering to the rules and procedures of programmes such as the FCPF or UN-REDD, but rather inviting partners (including bilateral and multilateral approaches) to support and complement the national approach that is being implemented. Up to now, existing REDD+ readiness support programmes like FCPF and UN-REDD have struggled to find a way through which to respect and support countries that choose their own approach (such as Gabon), as it does not ‘fit’ their established rules and procedures. Adopting a ‘different’ path for REDD+ readiness (such as in Gabon) is part of what should be considered as a country's ‘national circumstances’ (a fundamental concept in the REDD+, and indeed UNFCCC COP, Decisions). In light of the need to respect countries’ sovereignty and not exclude countries that choose a different approach to engage in REDD+ readiness, financing (especially of the multilateral type) for REDD+ readiness and capacity building for REDD+ M&MRV should become more flexible in the future. This could potentially be expressed in the form of two (or more) pathways to REDD+, with adapted rules and procedures for access to finance depending on the pathway selected by the country. Such programmes should review their rules and procedures to accommodate national circumstances and choices.

It is apparent that most financing is flowing into smaller scale projects rather than comprehensive strategic REDD+ national programmes. This was also established by Karsenty [15] for financing provided by the Congo Basin Forest Fund, leading to the observation that international financing for REDD+ is insufficiently focused on supporting large-scale strategic programmes linked to emerging national and sub-national REDD+ strategies. One of, or a combination of, the following four factors could explain this finding. First, it could be related to the points raised above about countries struggling to access REDD+ readiness financing at the national level through programmes such as the FCPF and UN-REDD or the Congo Basin Forest Fund. Second, projects at the sub-national level are often driven by international NGOs in the region, who have more capacity and experience to access financing and deal with the rules and procedures of various financing bodies. Third, the strategy of some countries (although not explicitly stated in any of the in-country visits) may be to test REDD+ readiness at the project scale before deciding whether or not to engage at the national level. Finally, as there is still some uncertainty concerning the positive incentives that will be received as part of REDD+, some countries may simply remain hesitant to make major changes at the national level to accommodate REDD+ readiness before the rules are formalized at the international level.

The less straightforward relationship in terms of financing and M&MRV is not entirely surprising as M&MRV largely builds on pre-existing activities and capacities in countries, such as national forest inventories, national communications to the UNFCCC, and forest and land-use mapping. This makes it more difficult to assess the impact and efficiency of M&MRV funding. The impact of M&MRV readiness financing as a component of REDD+ readiness financing is also difficult to measure. We believe that, based on the analysis presented in this paper and using this paper as a basis, in a few years time one will be better able to evaluate the progress made in the COMIFAC countries in terms of M&MRV capacity for REDD+ readiness.

### (b) REDD+ and measurement, reporting and verification readiness status in the countries

**Figure 2.**
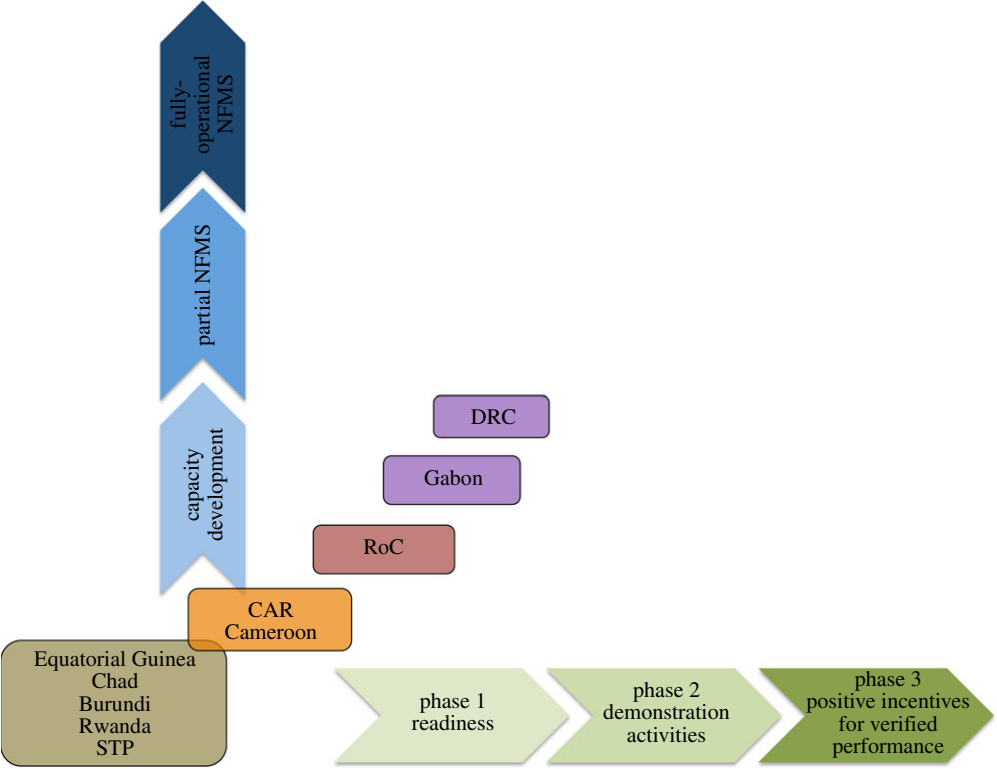
Status of countries in the REDD+ and M&MRV phases.

Another observation is that distinct country groupings appear in terms of REDD+ and M&MRV readiness status. Figure 2 illustrates four groups: DRC and Gabon in a leading group; RoC in a middle-advanced group; Cameroon and CAR in a middle less-advanced group; and Equatorial Guinea, Chad, Burundi, Rwanda and STP in a fourth group (least advanced). This grouping is based on expert knowledge, inputs by various stakeholders and qualitative data and provides an important overview of the status of REDD+ and M&MRV in the region. This roughly corresponds to the countries’ status in [17], although they did not specifically assess countries’ M&MRV as a component of REDD+ readiness status. The status of REDD+ in countries appears strongly, but not exclusively, linked with financing received.

#### (i) Group 1

For the two leading countries, a separate analysis is necessary. Gabon has a generally well-organized political structure and an outwardly positive stance towards REDD+ at the highest government level, as well as towards conservation-oriented activities in general. This has been especially true over the past 10–15 years and may have permitted the establishment of a leadership stratum with a good understanding of the potential that REDD+ holds for leveraging options for a low-carbon development pathway. Generally speaking, Gabon has a strong state, which rests on a relatively higher level of wealth in the country, when compared with other Central African countries. This may enable the Gabonese government to better address development on a basis of careful planning. Based on this strength, the Gabonese government is in a position to better negotiate with donors and impose its sovereign will. In this case, the state is proactive from the top down and all relevant sections of government are made aware of the process.

DRC does not have the level of individual wealth that Gabon may have, however, it holds the largest portion of resources (forest and other) in the region and probably Africa. As such, the country may be considered as of high political interest for most bilateral cooperation agencies, but also for multilateral systems seeking visibility in the region, which may be easier to gain through activities in a single country of very large size rather than several projects in multiple countries. In addition to this, DRC now rests on a group of well advised and informed key political actors that have understood the potential of REDD+ to leverage financing and are able to use this in the interest of the country to acquire comparatively more funds than other countries. In this instance, it is unclear whether the state is proactive from the top down or whether the individual influence of the ministries concerned is the primary driver. A further hypothesis for the increased funding level may simply rest on the sheer size of the country, where it is possible to inject large-scale investments into provinces that have surface areas that exceed the size of other mid-sized countries in the region.

#### (ii) Group 2

The RoC is in a middle-advanced group. This may reflect the influence of a large-scale effort to regulate the forestry sector over the past 5–10 years (e.g. through Forest Stewardship Council certification of forests), which may be resulting in increased awareness and a proactive stance in the forestry sector. Nevertheless, it is possible that this position lies only in responsible ministries rather than whole government structure. However, overall, the country's awareness remains limited and generally it appears that forestry-related activities might dominate the debate. A recent mining boom in this country may cast a shadow over the process as increasing interest may turn towards the mining sector before the potential of REDD+ has been fully explored.

#### (iii) Group 3

For the third group of countries, comprising Cameroon and CAR, the group is probably a consequence of weaker government institutions and lower awareness of REDD+ and the UNFCCC process in general, limited to officials appointed to the positions attached to the UNFCCC negotiations. An entrainment effect of the knowledge and influence of those officials in the government has not been reached as yet and further strengthening of government institutions is required to do so. Cameroon is more typical of this position as it is more politically stable and more likely to attract foreign investment than CAR.

#### (iv) Group 4

The fourth group of countries (Chad, Equatorial Guinea, Burundi, Rwanda and Sao Tome and Principe) represents the least advanced countries, with the exception of Rwanda which is economically more advanced than the other four countries. Excluding Chad, the other four countries are small and would generally be considered forest-poor. Other than Rwanda, these countries, owing to their low levels of economic development, have multiple other national development priorities and as a result the concept of REDD+ is generally poorly grasped by the relevant officials. These countries have therefore not been in a position to consider the options of applying REDD+ in their national circumstances as part of their national economic development strategies. In this group, it is highly probable that Rwanda, once it has been provided with a suitable awareness raising campaign, will shift to the ‘leading group’, owing to its strong government structure being able to leverage external expertise for specific purposes and objectives, such as REDD+.

### (c) Moving ahead

Unlike other regions, the COMIFAC region appears to have a number of enabling conditions in place for REDD+ and M&MRV to facilitate a tangible readiness opportunity and action, through a country-driven approach overarched by regional coherence. The following regional advantages were identified in the working groups during the kick-off meeting in N'Djamena (September 2012):

— Regional level political institution to enhance collaboration on forests (COMIFAC and its Executive Secretariat).
— A specific fund (i.e. the Congo Basin Forest Fund) to finance national and regional forest-related projects.
— COMIFAC's technical arm (OFAC), which is able to facilitate knowledge sharing and regional technical capacity building.
— A regional vision aligned to the objectives of REDD+.
— Countries currently not yet engaged in the REDD+ readiness process have expressed their wish to do so and learn from the experiences from other countries in the region.

A point noteworthy in its absence is the lack of information submitted by the private sector active in the Congo Basin on the voluntary database. A number of mining, logging, agricultural and industrial concerns of considerable political and financial importance at both the national and international levels are active in the region and represent a potentially significant driver of development [18]. This will have a direct bearing on REDD+ implementation, as it is likely to affect deforestation and forest degradation. The absence of any information from the private sector on REDD+ engagement could indicate either a single-sector approach to implementation, with cross-sectorial considerations and action driven by government (as in Gabon), or a lack of interest and action from REDD+ actors to engage the private sector in a meaningful way. In both instances, there is a strong case for better understanding this lack of participation.

## 5. Conclusion

We draw five key conclusions: (i) a significant amount of REDD+ financing flows into the Congo Basin (almost half of the REDD+ financing for the African continent); (ii) across the countries, there is an important disequilibrium in terms of REDD+ and M&MRV readiness financing, political engagement, comprehension and capacity, which also appears to be a key barrier to countries receiving equal resources; (iii) most financing appears to go to smaller scale REDD+ projects; (iv) four distinct country groups in terms of REDD+ and M&MRV status are identified; and (v) the Congo Basin has a distinct opportunity to develop a specific REDD+ financing window for large-scale and more targeted national REDD+ readiness support through a fund for the region (such as the Congo Basin Forest Fund).

Financing for REDD+ and M&MRV readiness and capacity building and a country having completed the ‘readiness phase’ is not a straightforward positive correlation. Financing or greater financing for REDD+ readiness alone is not necessarily a determinant of readiness ‘success’. Financing for REDD+ and M&MRV readiness is clearly only a first step in the REDD+ process, as the architecture for REDD+ implementation is based on three phases. As countries move through these phases, today's readiness efforts should lead to financing that becomes more reliable and based on results, such as is already the case in, for example, Brazil [19] and Guyana [20]—two countries that are receiving predictable funding based on results achieved.

We have shown that countries need to be able to absorb readiness financing, build awareness of the process for adequate stakeholder engagement and develop the capacity to understand COP decisions and IPCC guidance in order to undertake the steps that are needed for implementation. In this article, we argue that a more targeted and flexible approach to financing is needed, combined with a greater focus on understanding countries’ existing capacities and the ways in which they would like to engage with and implement REDD+. When REDD+ readiness financing is available, technical institutions should tailor the available financing in collaboration with country institutions for targeted capacity building, based on an analysis of what already exists—as presented in this paper. The proposal outlined in the discussion for a step-wise approach with smaller, more targeted financial REDD+ readiness support would allow countries with low institutional capacity to engage more actively and effectively; and to receive further support based on results and lessons learned through the step-wise approach.

The analysis presented in this paper provides a first expert insight that can be used by countries and financing bodies to engage in a dialogue on how to more effectively target REDD+ and M&MRV readiness financing and capacity building at COMIFAC countries. An important area for further study is for a similar analysis to be undertaken in a few years time (e.g. in 2018/2020 to coincide with the inception of a new agreement on climate change under the UNFCCC) to evaluate the progress made by the COMIFAC countries, using this first analysis as a basis, to further the REDD+ readiness lesson learning process.

## Endnotes

1 Reducing emissions from deforestation, reducing emissions from forest degradation, conservation of forest carbon stocks, sustainable management of forests and enhancement of forest carbon stocks [1].

2 ‘Also affirming the need to promote broad country participation in all phases described in paragraph 73 of 1/CP.16 including through the provision of support that takes into account existing capacities’ [5].

3 *‘*In order to promote progress to this end, each of these Parties shall communicate, within six months of the entry into force of the Convention for it and periodically thereafter, and in accordance with Article 12, detailed information on its policies and measures referred to in subparagraph (a) above, as well as on its resulting projected anthropogenic emissions by sources and removals by sinks of greenhouse gases*’.*

4 *‘*Develop, periodically update, publish and make available to the Conference of the Parties, in accordance with Article 12, national inventories of anthropogenic emissions by sources and removals by sinks of all greenhouse gases not controlled by the Montreal Protocol, using comparable methodologies to be agreed upon by the Conference of the Parties.*’*

5 Jérôme Gaugris, Pascale Janvier, Danilo Mollicone and Danae Maniatis.

6 Funding provided by a group of countries through one or more institutions/organizations/programmes, e.g. the World Bank.

7 Funding provided by one country to another country of organization.
